# Supplementary material for: The Neutrophil-to-Lymphocyte and Platelet-to-Lymphocyte Ratios Predict Reperfusion and Prognosis after Endovascular Treatment of Acute Ischemic Stroke
Source: J Pers Med. 2021 Jul 22;11(8):696. doi: 10.3390/jpm11080696 (PMC8399654; doi:10.3390/jpm11080696)

Supplementary Table S1. logistic regression analysis showing effect of NLR and PLR on unsuccessful reperfusion.

	aOR	95% CI		aOR	95% CI
Age	1.03	0.998-1.06	Age	1.03	1.002-1.06
Male	0.62	0.31-1.22	Male	0.64	0.33-1.25
NIHSS	1.02	0.097-1.09	NIHSS	1.03	0.98-1.09
Stroke mechanism			Stroke mechanism		
LAA			LAA		
CE	0.38	0.16-0.90	CE	0.34	0.15-0.80
Others	0.60	0.24-1.55	Others	0.73	0.31-1.78
Previous use of statin	0.24	0.08-0.73	Previous use of statin	0.27	0.10-0.78
Collateral status	0.98	0.64-1.48	Collateral status	0.98	0.65-1.48
WBC	0.95	0.85-1.05	WBC	1.03	0.94-1.12
LDL	1.004	0.99-1.01	LDL	1.003	0.99-1.01
HbA1c	0.79	0.57-1.09	HbA1c	0.77	0.56-1.06
platelet	1.00	1.001-1.01	platelet	1.001	0.998-1.01
NLR	1.11	1.04-1.19	PLR	1.004	1.001-1.01

Supplementary Table S2. logistic regression analysis showing effect of tertile of NLR and PLR on unsuccessful reperfusion.

	aOR	95% CI		aOR	95% CI
Age	1.03	1.003-1.06	Age	1.03	1.002-1.06
Male	0.61	0.31-1.20	Male	0.60	0.31-1.18
NIHSS	1.04	0.99-1.10	NIHSS	1.04	0.99-1.10
Stroke mechanism			Stroke mechanism		
LAA	0.35	0.15-0.83	LAA	0.37	0.16-0.86
CE	0.70	0.29-1.74	CE	0.74	0.30-1.83
Others	0.32	0.11-0.88	Others	0.31	0.11-0.88
Previous use of statin	0.999	0.66-1.52	Previous use of statin	1.02	0.67-1.55
Collateral status	1.01	0.92-1.10	Collateral status	1.04	0.95-1.13
WBC	1.003	0.99-1.01	WBC	1.004	0.99-1.01
LDL	0.76	0.55-1.05	LDL	0.75	0.54-1.04
HbA1c	1.003	0.999-1.01	HbA1c	1.00	0.998-1.01
Platelet					
NLR T1	1.33	0.56-3.12	PLR T1	2.56	1.06-6.22
NLR T2	2.51	1.11-5.70	PLR T2	2.84	1.15-6.99
NLR T3	1.03	1.003-1.06	PLR T3	1.03	1.002-1.06

Supplementary Table S3. logistic regression analysis showing effect of NLR and PLR on 3-month mRS 3 to 6.

	aOR	95% CI		aOR	95% CI
Age	1.05	1.02-1.07	Age	1.05	1.02-1.07
Male	0.72	0.38-1.38	Male	0.67	0.35-1.29
NIHSS	1.10	1.04-1.16	NIHSS	1.11	1.05-1.17
Stroke mechanism			Stroke mechanism		
LAA			LAA		
CE	0.30	0.13-0.68	CE	0.28	0.12-0.64
Others	0.60	0.24-1.47	Others	0.60	0.24-1.49
Previous use of statin	0.92	0.43-1.99	Previous use of statin	0.96	0.44-2.10
Collateral status	0.45	0.31-0.65	Collateral status	0.44	0.30-0.65
WBC	1.02	0.91-1.14	WBC	1.10	0.98-1.23
LDL	0.999	0.99-1.01	LDL	1.00	0.99-1.01
HbA1c	1.30	0.98-1.72	HbA1c	1.33	0.996-1.77
platelet	1.001	0.997-1.01	platelet	0.997	0.99-1.001
NLR	1.20	1.06-1.35	PLR	1.01	1.004-1.02

Supplementary Table S4. logistic regression analysis showing effect of tertile of NLR and PLR on 3-month mRS 3 to 6.

	aOR	95% CI		aOR	95% CI
Age	1.05	1.02-1.07	Age	1.05	1.02-1.07
Male	0.66	0.35-1.27	Male	0.66	0.35-1.25
NIHSS	1.11	1.05-1.16	NIHSS	1.10	1.05-1.16
Stroke mechanism			Stroke mechanism		
LAA			LAA		
CE	0.31	0.14-0.72	CE	0.29	0.13-0.65
Others	0.71	0.29-1.75	Others	0.67	0.28-1.65
Previous use of statin	0.89	0.41-1.94	Previous use of statin	0.91	0.43-1.95
Collateral status	0.460	0.32-0.68	Collateral status	0.44	0.30-0.64
WBC	1.03	0.93-1.15	WBC	1.09	0.98-1.22
LDL	0.999	0.99-1.01	LDL	1.000	0.99-1.01
HbA1c	1.31	0.996-1.73	HbA1c	1.27	0.97-1.67
Platelet	1.001	0.997-1.01		0.998	0.99-1.002
NLR T1			PLR T1		
NLR T2	2.32	1.15-4.70	PLR T2	1.52	0.75-3.09
NLR T3	3.67	1.67-8.06	PLR T3	2.59	1.20-5.55

Supplementary Table S5. Linear regression analysis showing the correlation between NLR and infarct volume (Total subjects and subjects with infarct of anterior circulation only)

	B	SE B	β	p	R	R^2
Constant	12.62	51.45		0.81	0.41	0.17
Age	0.04	0.39	0.01	0.92		
Male	-17.27	10.25	-0.01	0.09		
NIHSS	3.40	0.82	0.24	0.00		
Stroke mechanism	-6.16	7.22	-0.05	0.39		
Previous use of statin	-1.54	12.28	-0.01	0.90		
Collateral status	-10.72	5.53	-0.12	0.05		
WBC	1.42	1.62	0.06	0.38		
Platelet	0.06	0.06	0.06	0.34		
LDL	0.06	0.15	0.02	0.70		
HbA1c	-0.97	3.91	-0.01	0.80		
NLR	2.55	0.87	0.19	0.004		
anterior circulation only						
	B	SE B	β	p	R	R^2
Constant	6.614	57.50		0.91	0.41	0.17
Age	-0.10	0.43	-0.02	0.81		
Male	-18.59	11.24	-0.11	0.10		
NIHSS	3.73	0.94	0.24	0.00		
Stroke mechanism	-6.04	7.90	-0.05	0.45		
Previous use of statin	-0.16	13.16	-0.001	0.99		
Collateral status	-10.91	6.00	-0.11	0.07		
WBC	1.52	1.69	0.06	0.37		
Platelet	0.11	0.08	0.09	0.16		
LDL	0.10	0.16	0.04	0.55		
HbA1c	-1.17	4.07	-0.02	0.77		
NLR	2.58	0.97	0.18	0.01		

Supplementary Table S6. Linear regression analysis showing the correlation between PLR and infarct volume (Total subjects and subjects with infarct of anterior circulation only).

	B	SE B	β	p	R	R^2
Constant	0.83	51.74		0.99	0.40	0.16
Age	0.08	0.39	0.013	0.84		
Male	-16.85	10.32	-0.10	0.10		
NIHSS	3.56	0.82	0.25	<0.001		
Stroke mechanism	-4.60	7.25	-0.04	0.53		
Previous use of statin	-0.50	12.35	-0.002	0.97		
Collateral status	-9.85	5.58	-0.11	0.08		
WBC	3.30	1.44	0.14	0.02		
Platelet	-0.01	0.06	-0.01	0.84		
LDL	0.05	0.15	0.02	0.74		
HbA1c	-1.60	3.92	-0.02	0.68		
PLR	0.11	0.047	0.14	0.02		
anterior circulation only						
	B	SE B	β	p	R	R^2
Constant	-8.58	58.01		0.88	0.39	0.15
Age	-0.05	0.44	-0.01	0.92		
Male	-17.12	11.35	-0.10	0.13		
NIHSS	3.95	0.95	0.26	0.00		
Stroke mechanism	-4.20	7.95	-0.03	0.60		
Previous use of statin	1.82	13.28	0.01	0.89		
Collateral status	-10.59	6.11	-0.11	0.08		
WBC	3.21	1.58	0.13	0.04		
Platelet	0.07	0.08	0.06	0.40		
LDL	0.09	0.16	0.04	0.57		
HbA1c	-1.51	4.12	-0.02	0.71		
PLR	0.07	0.05	0.09	0.17		

Supplementary Table S7. Logistic regression analysis showing impact of NLR and PLR on unsuccessful reperfusion in LAA patients.

	aOR	95% CI		aOR	95% CI
Age	1.03	0.98-1.08	Age	1.03	0.99-1.09
Male	1.23	0.29-5.17	Male	1.32	0.35-5.02
NIHSS	1.05	0.93-1.18	NIHSS	1.07	0.95-1.19
Previous use of antithrombotics	0.11	0.01-1.05	Previous use of antithrombotics	0.14	0.02-1.06
Collateral state	1.57	0.59-4.17	Collateral state	2.26	0.76-6.69
Platelet	1.01	0.999-1.01	Platelet	1.002	0.997-1.01
HbA1c	0.66	0.39-1.12	HbA1c	0.78	0.48-1.27
NLR	1.35	1.05-1.75	PLR	1.01	1.002-1.02

Supplementary Table S8. Logistic regression analysis showing impact of NLR and PLR on unsuccessful reperfusion in CE patients.

	aOR	95% CI		aOR	95% CI
Age	1.02	0.97-1.07	Age	1.02	0.97-1.08
Male	0.59	0.21-1.72	Male	0.57	0.19-1.54
NIHSS	0.97	0.89-1.06	NIHSS	0.99	0.91-1.07
WBC	0.93	0.76-1.13	WBC	1.02	0.86-1.21
Platelet	1.01	1.00-1.02	Platelet	1.004	0.997-1.01
HbA1c	0.77	0.49-1.22	HbA1c	0.77	0.48-1.25
CRP	1.02	1.22-1.05	CRP	1.02	1.001-1.04
NLR	1.17	1.04-1.32	PLR	1.004	0.999-1.01

Supplementary Figure S1. Flow chart of study

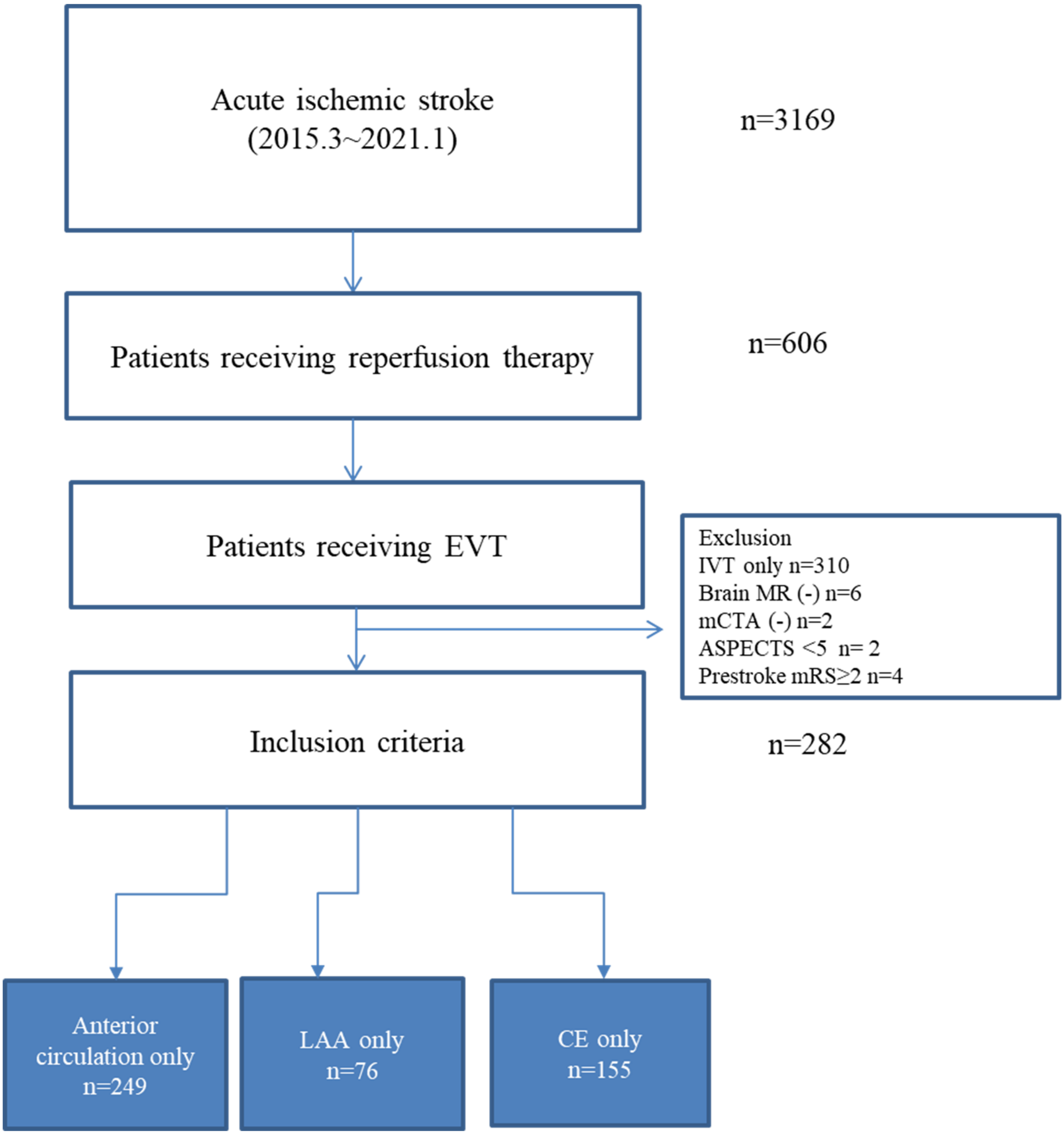

Supplement: Supplementary file 1 [file jpm-11-00696-s001.zip › jpm-1284412-SI.pdf]
